# Spatial, environmental and trophic niche partitioning by seabirds in a climate change hotspot

**DOI:** 10.1111/1365-2656.14245

**Published:** 2025-01-26

**Authors:** Yuri Niella, Dustin O'Hara, Ian Jonsen, David Slip, Lachlan Phillips, Rob Harcourt, Gemma Carroll

**Affiliations:** ^1^ School of Natural Sciences, Faculty of Science and Engineering Macquarie University Sydney New South Wales Australia; ^2^ Taronga Institute of Science and Learning Taronga Conservation Society Australia Mosman New South Wales Australia; ^3^ Environmental Defense Fund Seattle Washington USA; ^4^ Present address: Integrated Marine Observing System (IMOS), Animal Tracking Facility Sydney Institute of Marine Science Mosman New South Wales Australia

**Keywords:** climate change, East Australian Current (EAC), environmental niche, resource partitioning, satellite telemetry, seabirds, stable isotope analysis, trophic niche

## Abstract

1. For similar species to co‐occur in places where resources are limited, they need to adopt strategies that partition resources to reduce competition. Our understanding of the mechanisms behind resource partitioning among sympatric marine predators is evolving, but we lack a clear understanding of how environmental change is impacting these dynamics.
2. We investigated spatial and trophic resource partitioning among three sympatric seabirds with contrasting biological characteristics: greater crested terns *Thalasseus bergii* (efficient flyer, limited diver, and preference for high quality forage fish), little penguins *Eudyptula minor* (flightless, efficient diver, and preference for high quality forage fish) and silver gulls *Chroicocephalus novaehollandiae* (efficient flyer, limited diver and generalist diet). We investigated interannual variability in resource partitioning in relation to environmental variability in a climate change hotspot influenced by the warm and intensifying East Australian Current (EAC).
3. Sampling was conducted from 2012 to 2014 during the austral summer breeding season of seabirds at Montague Island, Australia. Daily seabird movements were monitored using GPS trackers and feather tissues were collected and processed for stable isotope analysis (δ^15^N and δ^13^C). Generalised Linear Mixed Models were used to assess how changes in oceanographic conditions influenced space use for each species. Schoener's D and Bayesian mixing models were used to respectively investigate the levels of yearly inter‐specific environmental and trophic niche overlaps.
4. Crested terns and little penguins were less likely to be observed in warm, saline EAC waters and crested terns and silver gulls had smaller foraging areas on days when more than 30% of available habitat was classified as EAC origin. All species preferred areas with low variability in sea surface temperature (<0.5°C). Terns and penguins occupied similar marine trophic levels, with penguins having larger isotopic niche spaces in 2014 when the EAC was more dominant in the study area. Gulls occupied the lowest trophic level, with the widest niche and lowest interannual variability in niche area.
5. As the EAC intensifies along the southeast coast of Australia under climate change, interspecific competition for resources may increase, with the greatest impacts on species like little penguins that have relatively restricted foraging ranges. This study suggests that species‐specific biological traits and behavioural plasticity should be accounted for when predicting the effects of climate change on marine species.

## Graphical abstract

As the East Australian Current intensifies along the southeast coast of Australia under climate change, intraspecific competition for resources by breeding seabirds may have greater impacts on species such as little penguins, that have more restricted foraging ranges. Our study suggests that species‐specific biological traits and behavioural plasticity should be accounted for when predicting the effects of climate change upon marine species.

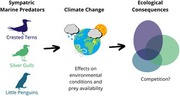

## 1 INTRODUCTION

How species coexist while sharing limited resources (i.e. ‘resource partitioning’) is a fundamental question in ecology (Schoener, 1974). The evolutionary pressure to reduce competition drives the formation of distinct ecological niches, wherein species differ in at least one of three main dimensions: time, habitat use or diet (Hearn et al., 2018). The segregation of species into diverse niches allows them to partition resources in ways that reduce overlapping demands and allow similar species to simultaneously meet their ecological requirements (Araújo & Guisan 2006; Chase & Leibold, 2003; Elton, 1927; Grinnell, 1917).

Marine megafauna provide unique insight into resource partitioning, with guilds of large, mobile predators with high energetic requirements competing for highly dynamic and patchily distributed prey (Robertson et al., 2014; Waite et al., 2012). For example, South American fur seals (*Arctocephalus australis*) have been found to target pelagic habitats and have more generalist diets than sympatric southern sea lions (*Otaria byronica*) which have more benthic and specialised diets, facilitating the coexistence of large populations of each species in the Falkland Islands (Riverón et al., 2021). In the southwest Indian Ocean, co‐occurring shark species use coastal waters at different times throughout the day, with some species also targeting different habitats (i.e. demersal vs pelagic) and prey, to reduce intra‐specific competition (Niella, Wiefels, et al., 2021; Trystram et al., 2017). Closely related species, such as great black‐backed (*Larus marinus*) and herring (*Larus argentatus*) gulls, can even occupy different trophic levels and focus on distinct habitats (i.e. marine vs. urban), enabling them to coexist (Lato et al., 2021).

While our understanding of resource partitioning in marine predators is increasing, little is yet known about how climate change will impact these dynamics. Biological traits such as body size and feeding strategy (i.e. generalist vs specialist diets) can influence how species respond to changes in ocean conditions (Sunday et al., 2015). And while the distributions of some marine megafauna are moving poleward to follow the displacement of thermal habitats and/or prey (Hill et al., 2016; Niella et al., 2020; Niella, Butcher, et al., 2021; Thorne & Nye, 2021), others have lower capacity to track resources (Carroll et al., 2024). Less mobile taxa, including those confined to nursery areas during early‐life development, those with a limited home range, or those that have specific habitat requirements for breeding, may be more vulnerable to the direct effects of ocean warming, and/or its indirect effects on the local availability of prey (Lear et al., 2019). For example, elevated temperatures during the 1997–1998 El Niño led to the extinction of many echinoderm species from a coral reef ecosystem in the Brazilian tropical northeastern coast (Attrill et al., 2004) and habitat reductions caused by ocean warming affect spawning success of some herring species (Petitgas et al., 2013). These species‐specific responses to climate change are likely to alter how sympatric predator species partition shifting resources, with potentially large impacts on competitive dynamics and the structure of ecological communities (Clewlow et al., 2019; Pickett et al., 2018).

The southeast coast of Australia is a climate change hotspot, with some latitudes warming up to three times faster than the global average (Hobday & Pecl, 2014, Varela et al., 2018, Malan et al., 2021). The rapid warming in this region is driven by intensification of the East Australian Current (EAC), a western boundary current that transports warm, nutrient‐depleted waters poleward. The EAC is a complex current, with its main jet separating from the coast at about 32° S, where it forms the eastward flowing EAC eastern extension (Oke et al., 2019) and where the southward propagating eddy field begins (Ridgway & Dunn, 2003). These eddies drive dynamic advections of warm water onto the continental shelf at weekly to seasonal timescales (Phillips, Malan, et al., 2022). These warm waters influence key biological processes in nearshore environments including phytoplankton productivity (Oke & Griffin, 2011), and survival and recruitment of fish larvae (Kasai et al., 2002), through the interplay between changes in water temperature and physical processes (Brassington et al., 2011).

Given such a rapid rate of warming, and the high seasonal and interannual variability in EAC dynamics, the southeast coast of Australia can be considered a natural laboratory to understand ecological outcomes under a magnitude of ocean warming that is expected to take decades in other regions (Pecl et al., 2014). A series of ecological disturbances have already been observed in this region due to EAC intensification (Suthers et al., 2011), including severe marine heatwaves causing disease outbreaks and elevating mortality rates at higher latitudes (Oliver et al., 2017), increased herbivory impacting coastal habitats due to shifting fish communities (Vergés et al., 2016) and higher temperatures reducing the foraging success of seabirds (Carroll et al., 2016). In addition, these warming waters have been responsible for changes in the distribution of marine predators throughout this region (Hill et al., 2016; Niella et al., 2020; Niella, Butcher, et al., 2021).

We assessed environmental influences on resource partitioning by three sympatric seabirds: greater crested terns (*Thalasseus bergii*), little penguins (*Eudyptula minor*) and silver gulls (*Chroicocephalus novaehollandiae*). These three species have overlapping spring/summer breeding seasons on Montague Island (Barunguba) in the path of the EAC, in one of the fastest warming coastal ecosystems in the world (Malan et al., 2021). This guild of predators provides an interesting model of how contrasting biological traits might influence shifts in patterns of resource partitioning in response to dynamic and changing environments. Greater crested terns are a shallow‐diving seabird that plunge to depths <1 m, specialising on high quality surface‐shoaling forage species such as anchovy (*Engraulis australis*; Quiring et al., 2021). Little penguins are flightless and pursue diverse prey, primarily schooling not only fish but also krill and jellyfish, usually at depths of <10 m but occasionally diving deeper than 50 m (Ropert‐Coudert et al., 2006). Silver gulls are surface feeders with high plasticity in both habitat use and diet, including using terrestrial habitat and anthropogenic subsidies including human food waste (Auman et al., 2011; Lenzi et al., 2019).

Because there is little temporal segregation of foraging effort among the three seabird species during the breeding season, we focused on illuminating differences in habitat selection and trophic niche, the remaining two dimensions of resource partitioning. We hypothesise that competition for resources will increase during years with higher influence of the warm, nutrient‐poor EAC, with potentially negative impacts on the ability of more specialised and less mobile species to shift their habitat or prey selection to cope with the changing availability of resources. Our study aims to shed light on the mechanisms underpinning responses to climate variability by marine predators with different movement and feeding strategies and explore the consequences for resource partitioning within ecological communities.

## 2 MATERIALS AND METHODS

### 2.1 Ethical approval

All work conducted was carried out under the authorisation of the Macquarie University Animal Ethics Committee (2011/054). The Committee approved all animal research protocols, which were undertaken in accordance with guidelines set out by Australian law. The work was authorised under Office of Environment and Heritage NSW Scientific Permits SL 100111 and SL 100746, and all relevant institutional and national guidelines for the care and use of animals were followed.

### 2.2 Study area and seabird sampling

**FIGURE 1:**
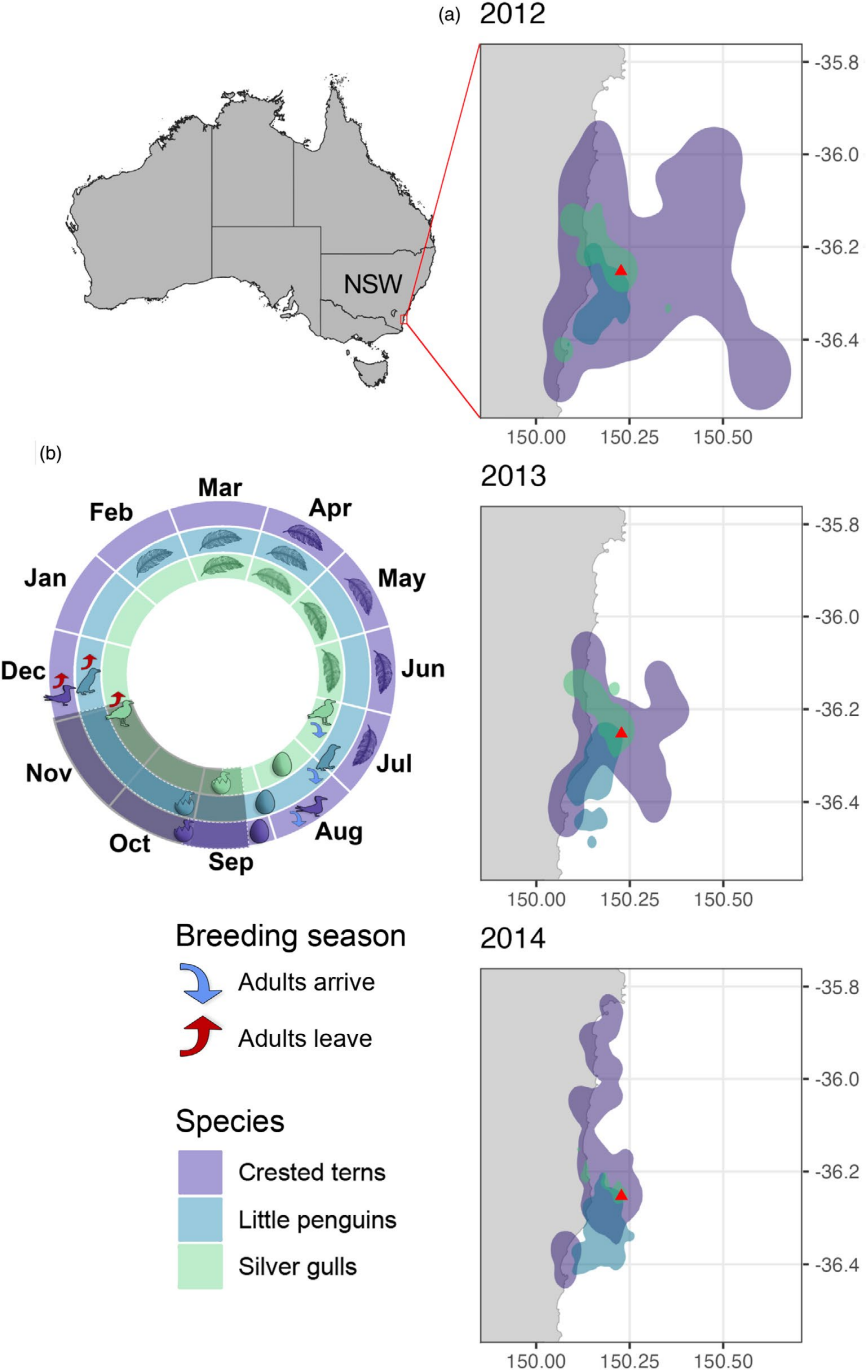
(a) Map of the study area showing the location of Montague Island in New South Wales (NSW) at the southeast coast of Australia, including the yearly 95% Kernel Utilization Distributions (KUD) from daily seabird tracks per species. The red triangles represent the location of Montague Island. For the number of individuals tracked from each species in each year, please refer to Table S1. (b) Seabird species‐specific annual breeding phenology including the dates adults arrive and leave Montague Island (Breading season), estimated mould periods (feather), when eggs are laid (eggs) and hatch (eggs with chicks) and when bird sampling was conducted between 2012 and 2014 (dark shaded area).

Montague Island is located off the southeast coast of New South Wales, Australia (Figure 1a), on the continental shelf. This region is strongly influenced by the EAC (Phillips et al., 2020; Phillips, Malan, et al., 2022), and is used by the three seabird species as a breeding site from July to December every year (Figure 1b). Crested terns and silver gulls that were incubating eggs in accessible nests were caught during the day using walk‐in or swing‐over traps. Walk‐in traps were comprised of a 50 × 40 cm wire cage with a narrow entrance placed over a nest, while swing‐over traps (also known as clap traps) consisted of a spring‐loaded, quick release net placed adjacent to a nest. Terns and gulls were all fitted with two Darvic colour leg bands to allow individual identification from a distance, and a GPS tag (Mobile Action Technology GT‐120, Taiwan, rehoused with a smaller battery in heat‐shrink tubing) was attached to feathers at the base of the tail using cloth‐backed tape (Tesa, Sydney). In 2013, six GPS devices on terns were lost in quick succession; so thereafter, devices on terns were attached on the back between the wings. The tag and the tape together weighed 14.1 g, 4.2% of mean tern body mass (335 g ± 4 SE) and 4.0% of mean gull body mass (351 g ± 6 SE). Birds were recaptured after 2–4 days to retrieve the GPS tags. Little penguins guarding small chicks were captured at night from artificial nest boxes and fitted with a CatTrack GPS tag (USA) that had been modified with epoxy resin to withstand pressure at depth (Carroll et al., 2016). Tags were sealed in heat‐shrink waterproof tubing and attached to the birds on the lower back with Tesa tape (Tesa, Sydney) to minimise drag or interference from birds' natural movements. Tags weighed 55 g, 5.0% of mean penguin body mass (1084 g ± 18 SE) in air and 1.2% in seawater. Tags were typically retrieved at the nest box the night following deployment.

A total of 163 birds were tracked between 2012 and 2014, including 32 terns, 34 silver gulls and 98 little penguins. The GPS tags were programmed to retrieve location fixes every 4 min in 2012 and 2013, and every 30 s in 2014. Stationary testing on the island with tags prior to deployment indicated an accuracy of 10–20 m and a precision of 20 m, and periods when tagged birds were incubating (in known locations) showed similar accuracy. Centroid locations were calculated using the raw seabird GPS coordinates with 12‐min intervals (i.e. 1 centroid from 3 locations for 2012 and 2013, and 1 centroid from 24 locations for 2014) to generate standardised tracks for all monitored individuals across the different sampling periods (Figure S1). The study area (Figure 1a) was considered as the total region used by all individuals tracked between 2012 and 2014 (Figure S1).

Feathers were collected from the back of each tracked bird upon GPS retrieval (see Section 2.7 for details on feather stable isotope analysis). The moulting schedule of silver gulls and greater crested terns is not well‐characterised in southeastern Australia, but gulls generally experience a prenuptial moult of body feathers (Dwight, 1925). Crested terns also moult into breeding plumage prior to the breeding season; however, there is no strong seasonal signal at the population scale (Dunlop, 1985). For both species, feathers are therefore likely to integrate information about the foraging ecology of these species over a period of at least several months prior to tracking, when they are more widely dispersed along the southeast Australian coast and may have different prey preferences (Quiring et al., 2021). Little penguins return to land following a period feeding at sea after breeding (February–April) and moult all their feathers. Penguin feathers are therefore informative about their foraging ecology during the relatively short time window preceding this total moult, approximately 6–8 months prior to tracking. All analyses were performed in the software R version 4.3.1.

### 2.3 Environmental variables

**FIGURE 2:**
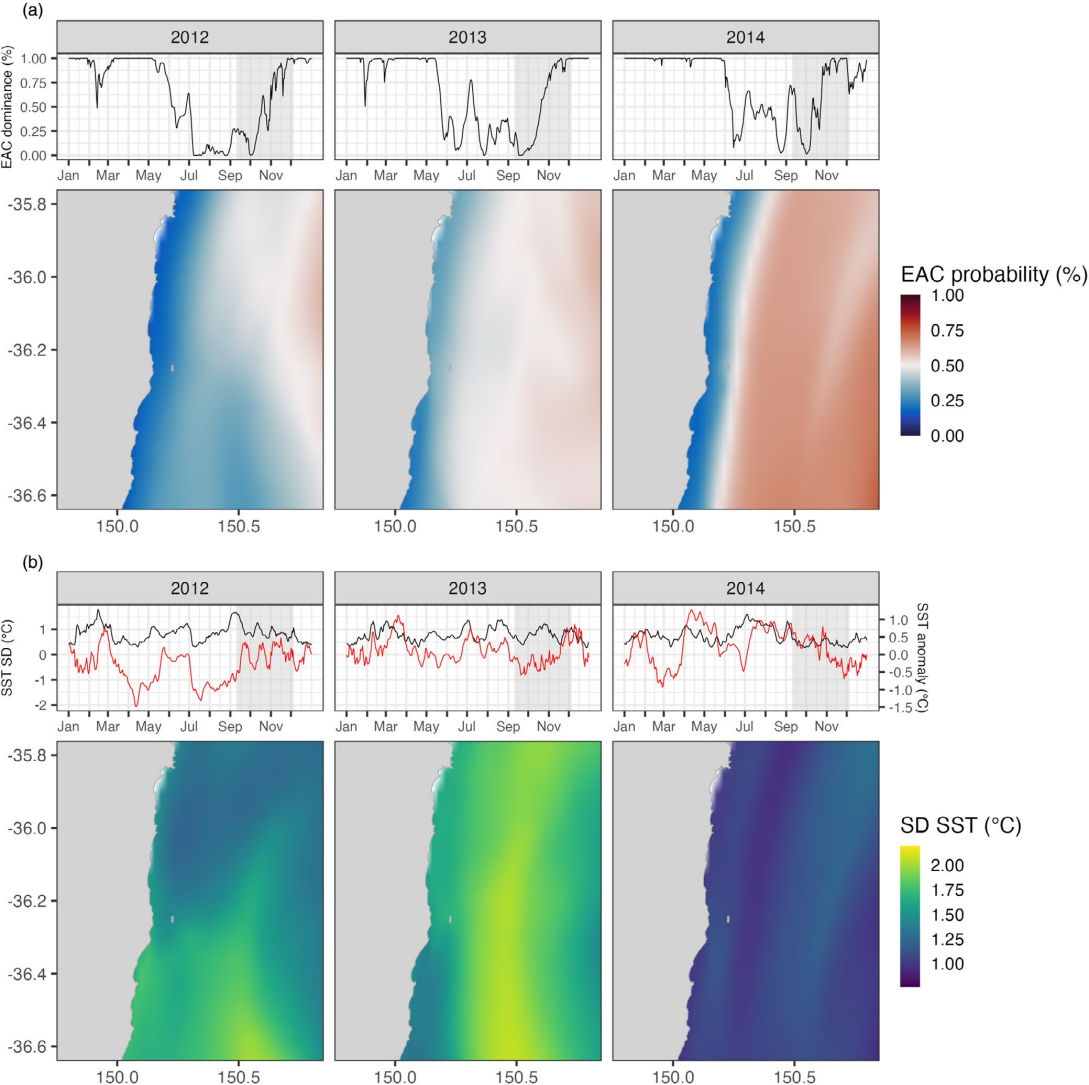
Yearly changes in the oceanographic conditions (a) East Australian Current (EAC) probability and (upper panel) dominance, and (b) sea surface temperature (SST) standard deviation (SD) within the study area and (upper panels) daily averages throughout the year (black lines) together with daily SST anomalies (red lines). Shaded areas (upper panels) represent the temporal windows (from 9 September to 5 December) when seabirds were sampled at Montague Island, NSW, Australia, used to calculate the average spatial distributions of each variable (lower panels).

Environmental data (rasters of 0.1° spatial resolution) were obtained from the Regional Ocean Modelling System for the southeast coast of Australia (Kerry & Roughan, 2020a). This is a hydrostatic equation developed to model ocean free‐surface flow on a curvilinear grid, validated using both remotely sensed and in‐situ observations (Kerry & Roughan, 2020b). Daily rasters of sea surface temperature (SST) were downloaded from this database for the period spanning 1 January 2011 and 31 December 2014 for the study area (36.6° S, 149.8° E to 35.8° S, 150.8° E). Since the EAC affects not only oceanographic but also ecological processes such as changes in seabird prey capture success linked to variations in SST (Carroll et al., 2016), its influence along the study region was also included in the analysis. A machine learning algorithm previously developed to track variability in the EAC was used to classify surface water in relation to their origin (hereafter referred to as the ‘EAC probability’ variable), in which waters of >0.5 probability were considered as being derived from tropical EAC source waters in the Coral Sea (Phillips et al., 2020). This algorithm was applied to ROMS surface data and results were output at the same grid scale (Figure 2a). For more details on the environmental data calculations, see Kerry and Roughan (2020a, 2020b) and Phillips et al. (2020).

The EAC is characterised by warm, tropical water (Li et al., 2022); thus EAC probability and SST were found to be highly correlated (Pearson's correlation; correlation coefficient = 0.9, *t* = 91.0, *p* < 0.001). Therefore, a standard deviation of SST (hereafter referred to as SST SD) variable was calculated daily for the study area, as each raster cell's standard deviation from daily mean SST values. SST SD was considered representative of habitat complexity within the study area, where cells with higher SST SD values represent areas where the water temperatures changed more (either cooling or warming) than areas with lower SST SD (Figure 2b). As EAC probability and SST SD were found to be only weakly correlated (correlation coefficient = −0.1, *t* = −7.4, *p* < 0.001), the two variables were simultaneously included in all analyses.

### 2.4 Proxies for environmental variation

Two metrics were used as proxies of interannual environmental variation in the study area. Both SST SD and EAC variation were compared between years, during the seabird sampling periods (Figure 2). Since waters with EAC probability >0.5 are considered to be derived from EAC source waters in the Coral Sea, according to the machine‐learning classification approach from Phillips et al. (2020), the percentage of raster cells with EAC probability >0.5 within the study area (hereafter referred to as EAC dominance) were calculated for each day of the sampling periods and its logarithm used for comparison purposes. The average of all SST SD cells was used as the daily values representative of this variable. Daily values of log EAC dominance and SST SD were then compared between years using Analysis of Variance (ANOVA) with post hoc Tukey tests.

### 2.5 Modelling seabird presence/absence and utilisation distribution areas

Seabird GPS tracks (Figure S1) were used to calculate daily species‐specific KUDs by pooling raw tracks from all individuals (Figure S2a). The 95% level KUDs were then used to extract only the daily EAC probability and SST SD values for the areas used by each species. Raster cells located within each 95% KUD were considered as presence, whereas the outside cells were classified as absences (Figure S2b,c). In addition, the respective number of individuals tracked from each species was included in analyses. The areas of species‐specific 95% KUDs (in km^2^) were calculated to be used as a proxy of daily seabird space use and yearly average space use was calculated for each species as the mean of daily 95% KUDs.

Due to the linear relationships found between our response variables and the environmental predictors tested, generalised linear mixed models (GLMM) were used to investigate patterns in how the environment influences seabird occurrence and space use. First, seabird occurrence was modelled using presence (one) and absence (zero) daily data obtained from the 95% KUDs as the response variable with a binomial response at both daily and yearly scales. Second, space use was modelled including the daily 95% KUD area as the response, which was log‐transformed to meet normality and modelled using Gaussian error distribution. The basic GLMM formula corresponded to:

$$y_{t}=\left(S*P_{t}\right)+\left(S*T_{t}\right)+1\;|\;t+\log_{10}N_{t}$$

Where, *y*_*t*_ represents the respective KUD occurrence or space use variable (i.e. presence/absence or KUD size area, respectively), the fixed terms are expressed as the interactions between seabird species (S) and the daily measurements of the environmental predictors EAC probability (*P*_*t*_) and SST SD (*T*_*t*_), including sampling day (*t*; continuous across different years) as a random factor to account for the species not being tracked during the exact same days in each year thus potentially reducing any residual temporal autocorrelation. Both models also included the logarithm of the daily number of individuals tracked (N) from each species as an offset to account for differences in sampling units, that is, different number of seabirds tracked on each day. Final models were visually inspected for a normal residual distribution.

### 2.6 Seabird niche overlaps

Different numbers of individuals from each seabird species were tracked in each year (Table S1). Therefore, a sensitivity analysis was conducted to ensure patterns would not be an artefact of sampling unit, for example, lower overlaps because fewer animals were tracked during a particular year. For this purpose, the lowest number of seabirds tracked (*N* = 8) was used (Table S1) to subsample the corresponding datasets 100 times with replacement, prior to conducting the niche overlap calculations. In each iteration, eight random animals from each species were selected from the tracking dataset for each sampling year. Environmental niche (see the following section for details) and KUD overlaps were calculated for each iteration and seabird species in pairs. KUDs and their overlaps were calculated using the adehabitatHR package (Calenge & Fortmann‐Roe, 2023). Variations in the amounts of overlap were compared for both metrics using an Analysis of Variance (ANOVA), including the variables species combination, year and resampling iteration. The ANOVA indicated that niche overlaps varied significantly as a function of species combination and year, but not as a function of resampling, for both metrics (Table S2). This suggests that the resampling approach effectively accounted for uncertainty due to the unbalanced interannual sampling.

### 2.7 Environmental niche

Schoener's D was used as the index of environmental niche similarity between pairs of species, for each year of satellite tracking data. This metric can estimate the similarity (as a percentage) in the niches occupied by two different species, accounting for multiple spatial and environmental parameters (Schoener, 1970). The approach included the variables: latitude, longitude, EAC probability and SST SD. Location data was first used to generate occurrence rasters with 0.05° latitude by longitude resolution for the resampled groups of seabird species (*N* = 8) for each year of tracking. These layers were then used to obtain daily rasterised data with the same spatial resolution as the EAC probability and SST SD variables for the cells occupied by each species. A principal component analysis (PCA) was then used to reduce the multidimensionality of the data (i.e. species occurrence and environmental variables), by combining the species occurrence rasters in pairs (one for each seabird and year) as response variables and including their corresponding sets of environmental rasters as explanatory variables (e.g. Broennimann et al., 2012). The density distributions derived from the first two PCA axes were then used to calculate Schoener's D as a representation of environmental niche overlap between each pair of species for each tracking year with the ecospat R package (Di Cola et al., 2017).

### 2.8 Seabird trophic niche

Upon GPS retrieval, a sample of 1–3 feathers was removed from the back of each tracked individual for stable isotope analysis. The feather samples were cleaned with a 2:1 chloroform: methanol mixture in order to remove both contaminants and the bird's own preen oil and then air‐dried for 48 h (April & William, 2006). A sample of 1 mg (±0.2 mg) was cut from each feather using stainless steel scissors and placed into a tin cup. Most of each sample was made up of rachis, taken from the intersection of the rachis and calamus in order to prevent contamination by blood and to account for potential within‐feather variation (Grecian et al., 2015). Analysis of feather stable carbon and nitrogen isotopes was conducted at the UC Davis Stable Isotope Facility (California, USA) via continuous flow isotope ratio mass spectrometry using a PDZ Europa ANCA‐GSL elemental analyser interfaced to a PDZ Europa 20–20 isotope ratio mass spectrometer (Sercon Ltd., Cheshire, United Kingdom). Isotope ratios are reported as δ‐values and expressed as ‰ according to the equation:

$$\delta X=\left[\left(\frac{R_{\text{sample}}}{R_{\text{standard}}}\right)-1\right]\times 1000$$

Where *X* is ^13^C or ^15^N, and *R*_sample_ is the corresponding ratio of ^13^C/^12^C or ^15^N/^14^N and *R*_standard_ is the ratio of the international standards V‐PDB (Vienna PeeDee Belemnite) and in air for carbon and nitrogen, respectively. The SD of multiple analyses of two laboratory standards is 0.2‰ for 13C and 0.3‰ for ^15^N (Votier et al., 2011).

Stable isotope values of δ^13^C (reflecting an inshore‐offshore gradient in foraging location) and δ^15^N (reflecting trophic position) were obtained from a total of 159 individuals from the three species during the study period (Table S1). It is important to note that some gull species may also access food sources from terrestrial origin (Lenzi et al., 2019), complicating comparisons with seabirds relying solely on marine prey and increasing the importance of complementary insights from tracking data (Mendes et al., 2018). Differences in isotopic composition between seabirds were assessed with Gaussian GLMMs for each element, including the effects of species as a candidate fixed effect and year as a random factor.

A Bayesian approach was used to estimate the yearly isotopic niche spaces including all the respective SIA samples from each species, using both carbon and nitrogen values with 100,000 posterior draws in the SIBER R package (Jackson et al., 2011). This framework is capable of overcoming limitations in community structure and niche occupancy comparisons when using simpler point‐estimate approaches that often ignore intra‐individual variability (Jackson et al., 2011).

## 3 RESULTS

### 3.1 Interannual changes in environmental conditions

The influence of the EAC during the seabird reproductive season varied between years (Figure 2a), with 2014 showing significantly higher dominance of EAC waters than either 2012 or 2013 (*F*‐value = 12.4, *p* < 0.001; Figure S3). 2014 also had the warmest ocean temperatures, with SST anomalies of around 0.5°C during September and October, and the lowest variability in SST as measured by SST SD. By contrast, 2012 was characterised by relatively low dominance of EAC waters in the study area (Figure 2a), both warm and cool daily surface temperature anomalies and higher spatial habitat complexity in the north of the study area (Figure 2b). 2013 had the coolest average daily surface temperatures and the highest spatial variability (~2°C) (ANOVA; *F*‐value = 55.2, *p* < 0.001).

### 3.2 Seabird interannual movement patterns

The tracked seabirds were found to use an area spanning ~4300 km^2^, with crested terns generally moving across wider distances from the colony (maximum = 46.4 km, mean = 7.4 ± 11.0 km) than little penguins (maximum = 29.2, mean = 9.6 ± 4.6 km) and silver gulls (maximum = 23.9, mean = 2.6 ± 5.0 km; Figure S1). The crested tern KUDs were consistently larger and extended further offshore, particularly in 2012 and 2013, than the other two species (Figure 1a). While silver gull 95% space use spanned primarily northwards from 2012 to 2014, little penguins moved mostly southwards from the colony (Figure 1a). Silver gulls showed consistent movements to a waste management facility located ~12 km northwest from Montague Island, particularly in 2012 and 2013 (Figure S4).

### 3.3 Environmental influences upon seabird space use

Both seabird occurrence (deviance explained = 60.1%) and size of 95% KUD areas (48.3%) were significantly influenced by the variables EAC probability and SST SD and were found not to vary across sampling days (Table 1). Crested terns and little penguins were found to prefer areas with <30% EAC probability (Figure S5a), and all species were observed to use mostly areas with <0.5°C variation in water temperatures (Figure S5b). Both crested terns and silver gulls used significantly smaller areas during days when the EAC probability was higher than 30% (Figure S5c), with all species found to move across larger areas when foraging habitat was less predictable, with SST SD between 0.3 and 1°C (Figure S5d).

**TABLE 1:** Generalised linear mixed models of daily seabird presence/absence and maximum distances travelled as a function of the interactions between seabird species and sea surface temperature (SST) standard deviation (SD) and East Australian Current (EAC) probability, and sampling day.

Models	Variables	Type	Est	SE	*t*	*p*
Presence/absence	Crested terns × EAC probability	Fixed	−4.62	1.81	−2.55	0.011*
	Little penguins × EAC probability	Fixed	−4.18	1.09	−3.84	<0.001*
	Silver gulls × EAC probability	Fixed	−6.60	5.24	−1.26	0.209
	Crested terns × SST SD	Fixed	−8.34	1.46	−5.69	<0.001*
	Little penguins × SST SD	Fixed	−9.66	1.57	−6.15	<0.001*
	Silver gulls × SST SD	Fixed	−21.89	4.92	−4.45	<0.001*
	Sampling day	Random				0.681
95% KUD area	Crested terns × EAC probability	Fixed	−0.70	0.37	−1.91	0.059
	Little penguins × EAC probability	Fixed	0.22	0.24	0.93	0.357
	Silver gulls × EAC probability	Fixed	−4.23	1.10	−3.83	<0.001*
	Crested terns × SST SD	Fixed	2.99	0.42	7.08	<0.001*
	Little penguins × SST SD	Fixed	1.01	0.32	3.13	0.002*
	Silver gulls × SST SD	Fixed	4.63	1.02	4.52	<0.001*
	Sampling day	Random				0.637

*Note*: Included are the corresponding variable types, coefficient estimates (Est), standard errors (SE), *t*‐values (*t*) and *p*‐values (*p*). Significant *p*‐values are highlighted (*).

### 3.4 Interannual variation in seabird trophic niche

**FIGURE 3:**
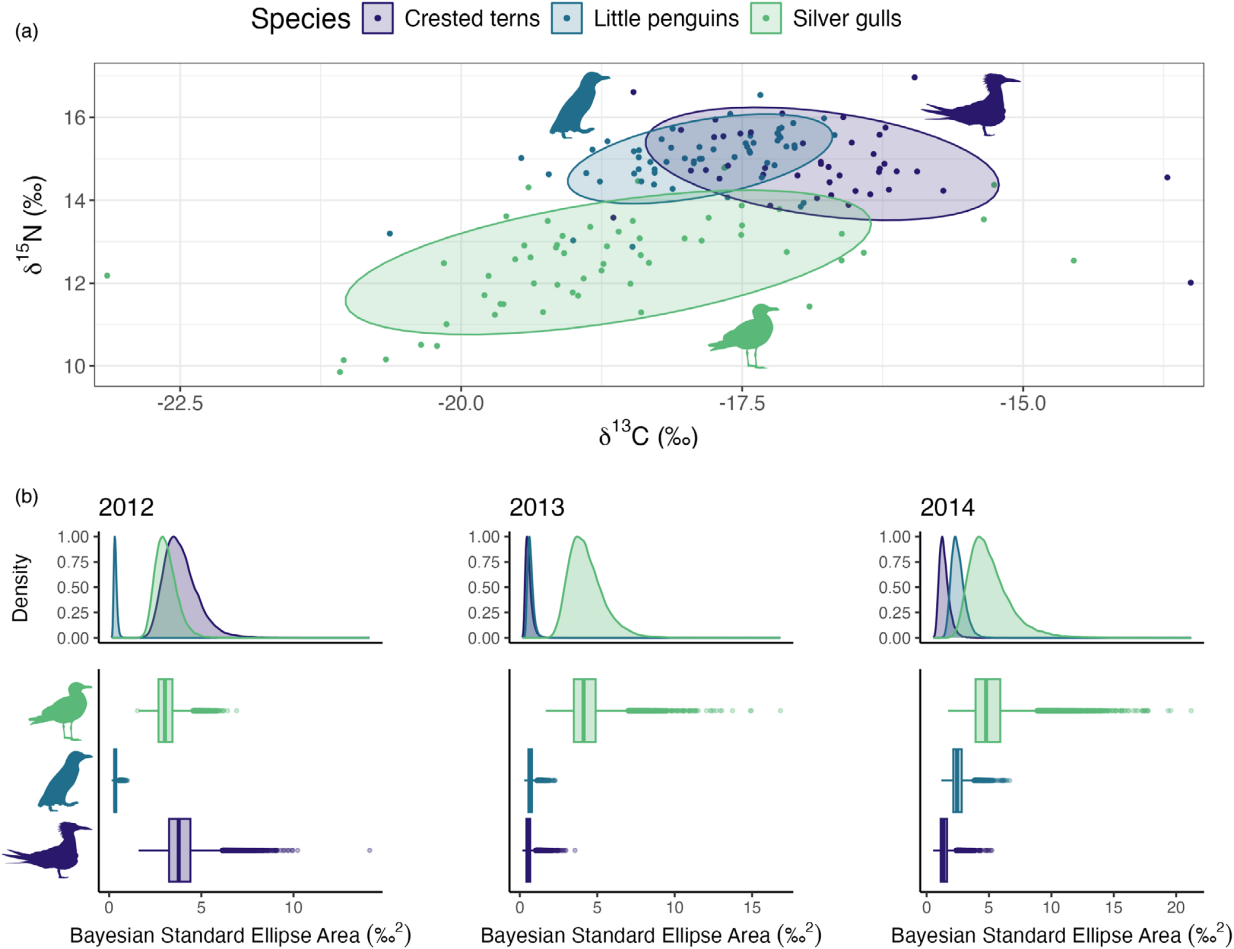
(a) Isotope biplots of δ^15^N and δ^13^C values by seabird species for all years combined. Points and circles represent every individual sampled and the species‐specific 95% normal confidence ellipses, respectively. (b) Yearly density plots from the Bayesian estimation of isotopic niche space (Bayesian Standard Ellipse Area) calculated for each seabird species. Density distributions (upper panels) represent the most frequently modelled isotopic niche spaces, and boxplots (lower panels) depict their respective median, inter‐quartile (boxes) and 95% ranges (lines), and outline (points) values.

The δ^13^C values were found to vary significantly across species but not between sampling years (deviance explained = 30.8%; Table 2). Crested terns and little penguins were found to have similar δ^15^N values which differed from the silver gulls, with significant interannual variation (deviance explained = 65.1%; Table 2). Crested terns and little penguins were found to have similar total trophic niches and to have little total overlap with the silver gulls (Figure 3a). The two species were also found to occupy higher marine trophic levels than silver gulls in all sampling years, with differences between them being particularly pronounced in 2012 and 2013 (Figure S6a). There was greater spatial variation in trophic niche among the silver gulls sampled in 2013 and little inter‐annual differences for the other two species (Figure S6b). Bayesian estimation of interannual isotopic niche space indicated that silver gulls had larger trophic niches than crested terns and little penguins, particularly in 2013 and 2014, with little variation across sampling years (Figure 3b). Higher interannual variation in trophic niche was found for little penguins and crested terns, with terns having larger trophic niches in 2012 and penguins having larger trophic niches in 2014, compared to the other years (Figure 3b).

**TABLE 2:** Generalised linear mixed models (GLMM) of ^13^C and ^15^N isotope variations as a function of the variables seabird species (crested terns, little penguins and silver gulls) and year.

Isotope	Variables	Type	Est	SE	*t*	*p*
^13^C	Crested terns	Fixed	−16.79	0.19	−86.43	<0.001*
	Little penguins	Fixed	−1.09	0.23	−4.79	<0.001*
	Silver gulls	Fixed	−1.90	0.23	−8.34	<0.001*
	Year	Random				0.934
^15^N	Crested terns	Fixed	457.22	159.65	2.86	0.004*
	Little penguins	Fixed	0.17	0.18	0.98	0.328
	Silver gulls	Fixed	−2.42	0.18	−13.53	<0.001*
	Year	Random				0.004*

*Note*: Included are the corresponding variable types, coefficient estimates (Est), standard errors (SE), *t*‐values (*t*), *p*‐values (*p*) and percentages of deviance explained (Dev.exp) of each model. Significant *p*‐values are highlighted (*).

### 3.5 Seabird interannual overlaps

**FIGURE 4:**
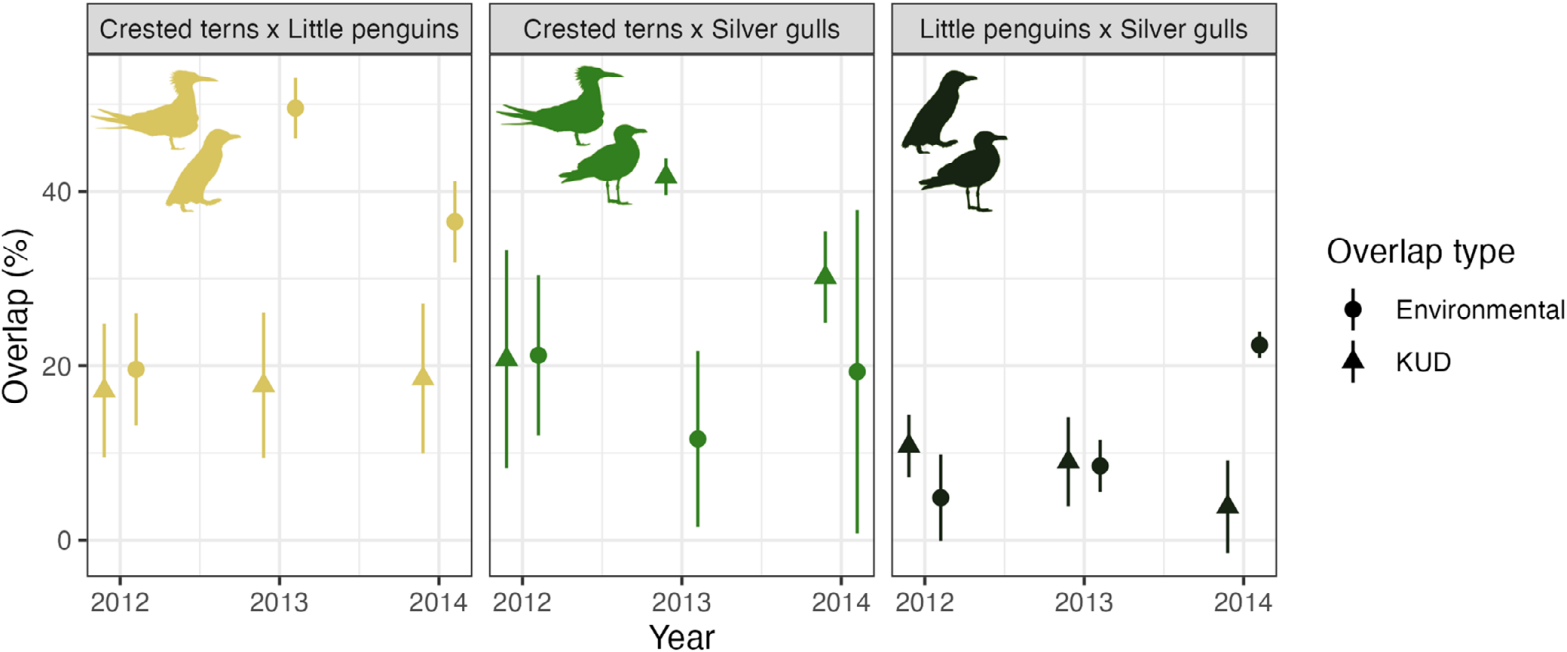
Yearly percentages of environmental niche (points) and KUD (triangles) overlaps between pairs of seabird species.

Changes in both environmental niche and KUD overlap between pairs of seabird species were found to vary significantly across the seabird species and during the study period (Table S2). While the environmental niche overlap between crested terns and little penguins increased almost twofold from 2012 to 2014, their KUD overlap remained constant between years (Figure 4). However, an overall increase in environmental niche overlap throughout the study period between crested terns and silver gulls, and between little penguins and silver gulls, was respectively followed by an increase and a decrease in their spatial (i.e. KUD) overlaps between years (Figure 4).

## 4 DISCUSSION

While our understanding of how sympatric animals coexist via resource partitioning is increasing, it remains unclear how such strategies might alter in space and time in response to changes in environmental conditions. In systems where food availability is highly variable, species are expected to show limited niche separation in periods of high food availability but increase resource partitioning when food availability is low and competition for resources is greater (Sabarros et al., 2012; Schoener, 1974). Using movement and trophic data, we explored how three co‐occurring seabird species partition resources in an ocean climate change hotspot in southeast Australia (Sunday et al., 2015). Our findings indicate that seabird resource partitioning in this system occurred across multiple scales and was likely influenced by both the gradients in environmental conditions and species‐specific biological traits. Gulls had the widest niche space reflecting their ability to forage in both terrestrial and marine environments. Penguins and terns showed strong overlap in trophic niche space despite their feather chemistry reflecting foraging conditions at different times of the year. However, little penguins had a consistently smaller realised trophic niche space and highly constrained foraging ranges relative to both flying seabirds, despite highly dynamic ocean conditions. These findings suggest that marine predators with constrained mobility, like little penguins, may have lower potential for adaptation in response to increasing ocean climate change impacts relative to their co‐occurring competitors.

Since the distribution of marine animals along the east coast of Australia is influenced by the EAC both directly via advection and access to suitable habitat, and indirectly via effects on prey availability, its recent intensification is leading to alterations in movement ecology of many species. This includes the tropicalisation of temperate reef communities (Bates et al., 2014; Vergés et al., 2016), changes in the timing and duration of migration seasonality (Champion et al., 2019) and predators spending more time at higher latitudes (Hazen et al., 2013; Niella et al., 2020; Niella, Butcher, et al., 2021). While our study spanned only 3 years, this period contained considerable environmental variability, with 2014 showing increased dominance of the warm EAC in the study area, with elevated sea surface temperatures and low spatial variability in thermal habitat. On the other hand, 2012 and 2013 showed much cooler and more variable conditions associated with greater influence of non‐EAC waters originating in the Tasman Sea (Phillips et al., 2020). Within this period, the three seabird species generally showed fine‐scale habitat preferences for cooler, non‐EAC waters where SST was more predictable, and expanded their foraging ranges when SST was less predictable. This highlights their sensitivity to oceanographic conditions associated with EAC dynamics at within‐season timescales, and the relative strengths of these relationships emphasise species‐specific abilities to adjust foraging distributions to seek out favourable habitat under adverse conditions.

### 4.1 Species‐specific responses to oceanographic variability

Species‐specific biological traits influencing responses to environmental variability include flight and diving ability, diet and ability to use anthropogenic habitats (Auman et al., 2011; Clewlow et al., 2019; Petalas et al., 2021; Pickett et al., 2018; Quiring et al., 2021; Ropert‐Coudert et al., 2006). Due to reduced foraging opportunities as a consequence of climate change, seabirds would probably need to travel further distances, dive to greater depths or even rely on human wastes in order to find food.

Crested terns had the greatest mobility of the three species, travelling further from the colony during each foraging trip than gulls or penguins. Although they generally foraged across the continental shelf, in 2014, when the EAC was furthest inshore, most dominant and least variable (Figure 2a), the terns hugged the coastline and extended their distribution much further north (Figure 1a), likely to avoid foraging in warmer EAC waters or possibly to take advantage of near coast upwelling events that can be driven by the advection of the EAC onto the shelf (Phillips, Malan, et al., 2022; Roughan & Middleton, 2004). Fast‐flying seabirds such as terns appear to have a greater ability to alter foraging location with changing environmental conditions, as they can potentially reach patches of resources further afield than swimming or shorter ranging seabirds (Cannell et al., 2012; Hamer et al., 2007), and may also target different prey types to reduce competition (Robertson et al., 2014). Such features could enable crested terns in this region to flexibly shift both their distribution and diet if ocean warming associated with EAC intensification continues to impact prey availability.

The low diving ability of terns limits their capacity to exploit prey that is distributed deeper in the water column. Terns forage by plunge‐diving to catch small coastal pelagic fishes, with a diet dominated by Australian anchovy (*Engraulis australis*) at the Montague Island colony (Quiring et al., 2021). Off the southeast coast of Australia, increased nearshore influence of the EAC reduced the number and density of potential prey aggregations, and increased their depth in the water column (Phillips, Malan, et al., 2022). The abundance and/or depth of high quality coastal pelagic prey species like anchovy may have been affected by increased dominance of the EAC throughout the late winter and spring of 2014, contributing to the downward shifts in tern trophic level (Figure S6b) and niche space (Figure 3b) observed between feathers collected in 2013 and 2014.

Little penguins are deeper divers than terns, and actively capture small fish, and occasionally squid, krill and jellyfish within the water column depending on availability (Cavallo et al., 2020). However, little penguins have a limited distribution (within 25 km from the colony) when they are guarding small chicks and make only single day foraging trips before returning to the nest. Little penguins showed a lower ability to increase their home range size or shift the location of their core foraging area in response to changes in foraging habitat compared to terns or gulls during this period of their breeding cycle. This constraint on their mobility is due to the slower speeds and higher energetic costs of swimming compared with flight, and a reduced ability to use long distance visual cues to identify high quality foraging opportunities across the seascape (Alexander, 2001). Penguins also occupied the smallest isotopic niche space, with the lowest variation in niche size between years, with narrow δ^13^C values reflecting their limited inshore foraging distribution and narrow δ^14^N values reflecting their preference for forage fish. Although little penguins have not been tracked from Montague Island during the non‐breeding season, data from other colonies suggest that most are resident year‐round (maximum foraging distance during extended foraging trips of 150 km; McCutcheon et al., 2011) but undertake intensive pre‐moult foraging trips to gain enough body condition for a 2–3‐week fast during the moult (Kowalczyk et al., 2015; Salton et al., 2015). Penguin feather isotopes therefore reflect foraging conditions from the region around Montague Island during approximately December to March, at a time when they have a strong drive to find a lot of high‐quality prey (Kowalczyk et al., 2015; Reinhold et al., 2022).

Little penguins are generally able to match the distribution of their prey within their foraging range (Carroll et al., 2017; Phillips, Carroll, et al., 2022) and use information about their prior prey capture experiences to inform their foraging site selection in the short term, while maintaining flexibility in response to prey availability and environmental conditions (Camprasse et al., 2017; Carroll et al., 2018). In one colony, higher breeding success has been associated with increases in foraging area and a broader diet niche following changes in river outflow, suggesting that foraging flexibility can have payoffs for fitness in some cases (Kowalczyk et al., 2015). However, during EAC‐dominated years when prey was either less abundant or less accessible, penguins breeding at Montague Island had lower prey capture success and reduced body condition (Carroll et al., 2016; Phillips, Malan, et al., 2022).

In Western Australia, complete breeding failure has been recorded under marine heatwave conditions associated with influxes of the warm Leeuwin Current (Cannell et al., 2012) and a population decline of 94% has been attributed to warming waters (Cannell, 2024). This suggests that their foraging plasticity has limits, and there may be critical environmental thresholds beyond which behavioural adaptation cannot compensate for poor foraging conditions, particularly during the breeding season when foraging range is constrained. Colonies like Montague Island may meet these limits sooner, where water temperatures are increasing rapidly (Malan et al., 2021), and where the spring/summer breeding season overlaps with the period of highest EAC dominance. Penguins at this colony also appear to be foraging at a significantly lower trophic level than birds at colonies further south (Montague Island δ^15^N = 14–16 c.f. Phillip Island δ^15^N = 18–22, Kowalczyk et al., 2015), suggesting that foraging opportunities in this region may already be of lower quality. Taken together, this suggests that of these three species, penguins may be the most vulnerable to population declines under accelerating climate change due to their lower behavioural flexibility in the face of poor foraging conditions, particularly during the breeding season when their foraging range is constrained by the fasting ability of their partner and offspring.

The higher foraging plasticity of silver gulls (Carrick & Murray, 1964; Wheeler & Watson, 1963), highlighted here by their larger trophic niche compared to the other two species, and use of the waste management facility, suggest that the species may be somewhat less impacted by the effects of ocean warming along this region. Lesser black‐backed gulls (*Larus fuscus*) also switch their diets from marine to land‐focused, to reduce intra‐specific competition for resources (Corman et al., 2016). Therefore, the higher use of anthropogenic subsidies derived from the waste management facility observed in 2012 and 2013 (Figure S3) could have been a strategy to reduce competition with terns and penguins in years with lower EAC influence and consequent higher prey availability.

### 4.2 Implications for overlap and resource partitioning under future climate change

Off the southeast coast of Australia, increased nearshore influence of the EAC has been shown to reduce the number and density of potential prey aggregations, and increase their depth in the water column (Phillips, Malan, et al., 2022). These environmental changes have implications for the ability of seabirds to match prey distribution across their foraging ranges (Carroll et al., 2016, 2017; Phillips, Carroll, et al., 2022) and are likely to influence inter‐specific patterns of trophic niche overlap between years. As the EAC strengthens, waters continue to warm, and extreme heatwave events become more common in this region (Bull et al., 2020; Elzahaby et al., 2021; Holbrook et al., 2020), resource competition is expected to increase among sympatric seabirds and may become a particular threat for less mobile species. While increases in competition between penguins and gulls is unlikely due to their different diets, competition for high quality, surface‐schooling prey may increase between penguins and terns when both are constrained to foraging near the colony. This could be mitigated to some degree by the greater mobility of terns, which can fly further from the colony and exploit foraging areas further up or down the coast in response to changing oceanographic conditions and prey availability. Due to their more limited mobility, little penguins would benefit from a thriving marine environment closer to the island to increase their foraging opportunities during their breeding season. Montague Island is located within a nature reserve, and effective management and protection of this region could help minimise the impacts of ocean warming for the species.

Combining multiple metrics of species interactions (i.e. trophic and spatial overlap) can help us better understand whether sympatric species can adapt to coexist under changing environmental conditions, or whether competition poses increasing threats to more vulnerable species. Accounting for species interactions is essential to accurately predict outcomes for both individual species and ecological communities under climate change and can inform ‘climate‐smart’ conservation strategies such as protected area network design and ecosystem‐based fisheries management.

## AUTHOR CONTRIBUTIONS

Yuri Niella, Dustin O'Hara, Ian Jonsen, Robert Harcourt and Gemma Carroll conceptualised the study. Yuri Niella analysed the data and together with Gemma Carroll wrote the first draft of the manuscript, with contributions from Dustin O'Hara. Dustin O'Hara, Lachlan Phillips, Dave Slip, Robert Harcourt and Gemma Carroll collected the data. All authors contributed to the final version of the text.

## CONFLICT OF INTEREST STATEMENT

The authors declare no conflicts of interest.

## Supporting information


**Table S1.** Number of individual seabirds tracked and sampled for feathers to be included in the stable isotope analyses (SIA), by year and species.
**Table S2.** Sensitivity test using an analysis of variance of environmental niche and KUD overlap percentage variations as a function of the respective random seabird individual resampling.
**Figure S1.** Raw seabird daily GPS tracks per species and year, including the respective number of animals tracked (N).
**Figure S2.** Schematic diagram exemplifying the use of the (A) daily 95% Kernel Utilization Distributions to mask (B) the respective layers of environmental data into (C) presence (area used by the animals) and absence (areas where the animals did not move into) raster layers.
**Figure S3.** Yearly distributions of oceanographic conditions sea surface temperature standard deviation (SST SD) and the logarithm of number of >0.5 EAC probability cells [log(EAC >0.5)] within the study area during the periods of seabird sampling (from 9 September to 5 December).
**Figure S4.** Silver gull tracks by year, showing the location of the local waste management facility (Brou Tip; red inset) used by the species.
**Figure S5.** Generalised linear mixed models of daily seabird presence/absence (top panel) and size of daily 95% Kernel Utilization Distribution (KUD) areas (bottom panel), including the significant effects of (A, C) East Australian Current (EAC) probability, and (B, D) sea surface temperature standard deviation (SST SD).
**Figure S6.** Yearly variations in (A) δ^15^N and (B) δ^13^C values per seabird species.

## Data Availability

Data available from the Zenodo Digital Repository https://zenodo.org/records/14611721 (Niella et al., 2025). http://doi.org/10.5281/zenodo.14611720.
