# Supplementary material for: Spatial, environmental and trophic niche partitioning by seabirds in a climate change hotspot
Source: J Anim Ecol. 2025 Jan 26;94(4):582–96. doi: 10.1111/1365-2656.14245 (PMC11962239; doi:10.1111/1365-2656.14245)
Supplement: Supplementary file 1 — Table S1. Number of individual seabirds tracked and sampled for feathers to be included in the stable isotope analyses (SIA), by year and species. Table S2. Sensitivity test using an analysis of variance of environmental niche and KUD overlap percentage variations as a function of the respective random seabird individual resampling. Figure S1. Raw seabird daily GPS tracks per species and year, including the respective number of animals tracked (N). Figure S2. Schematic diagram exemplifying the use of the (A) daily 95% Kernel Utilization Distributions to mask (B) the respective layers of environmental data into (C) presence (area used by the animals) and absence (areas where the animals did not move into) raster layers. Figure S3. Yearly distributions of oceanographic conditions sea surface temperature standard deviation (SST SD) and the logarithm of number of >0.5 EAC probability cells [log(EAC >0.5)] within the study area during the periods of seabird sampling (from 9 September to 5 December). Figure S4. Silver gull tracks by year, showing the location of the local waste management facility (Brou Tip; red inset) used by the species. Figure S5. Generalised linear mixed models of daily seabird presence/absence (top panel) and size of daily 95% Kernel Utilization Distribution (KUD) areas (bottom panel), including the significant effects of (A, C) East Australian Current (EAC) probability, and (B, D) sea surface temperature standard deviation (SST SD). Figure S6. Yearly variations in (A) δ15N and (B) δ13C values per seabird species. [file JANE-94-582-s001.pdf]

Spatial, environmental, and trophic niche partitioning by seabirds in a climate change hotspot

Supplementary Material

Table S1. Number of individual seabirds tracked and sampled for feathers to be included in the stable isotope analyses (SIA), by year and species.

Year	Species	Tracking (N)	SIA (N)
2012	Crested terns	30	20
	Little penguins	30	18
	Silver gulls	30	30
2013	Crested terns	8	9
	Little penguins	30	16
	Silver gulls	19	16
2014	Crested terns	13	14
	Little penguins	30	25
	Silver gulls	10	11

Table S2. Sensitivity test using an analysis of variance of environmental niche and KUD overlap percentage variations as a function of the respective random seabird individual resampling.

Niche type	Variable	Sum^2	$Mean^2$	F	p
Environmental	Species combination	8.916	4.458	419.82	<0.001
	Year	1.769	1.769	166.62	<0.001
	Resampling	0.001	0.001	0.11	0.743
KUD	Species combination	8.17	4.09	391.18	<0.001
	Year	0.10	0.10	10.02	0.002
	Resampling	0.02	0.02	1.49	0.221

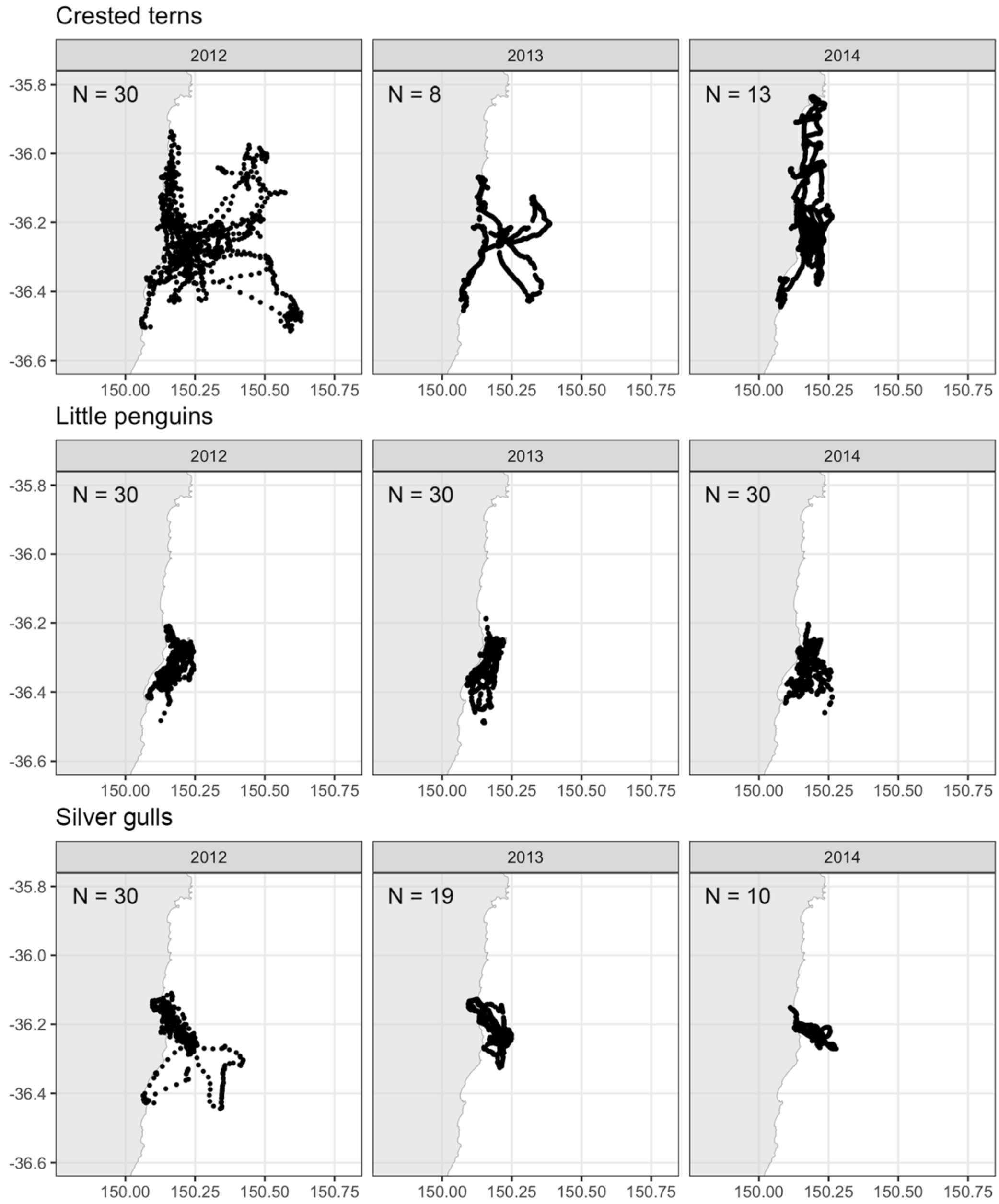

Figure S1. Raw seabird daily GPS tracks per species and year, including the respective number of animals tracked (N).

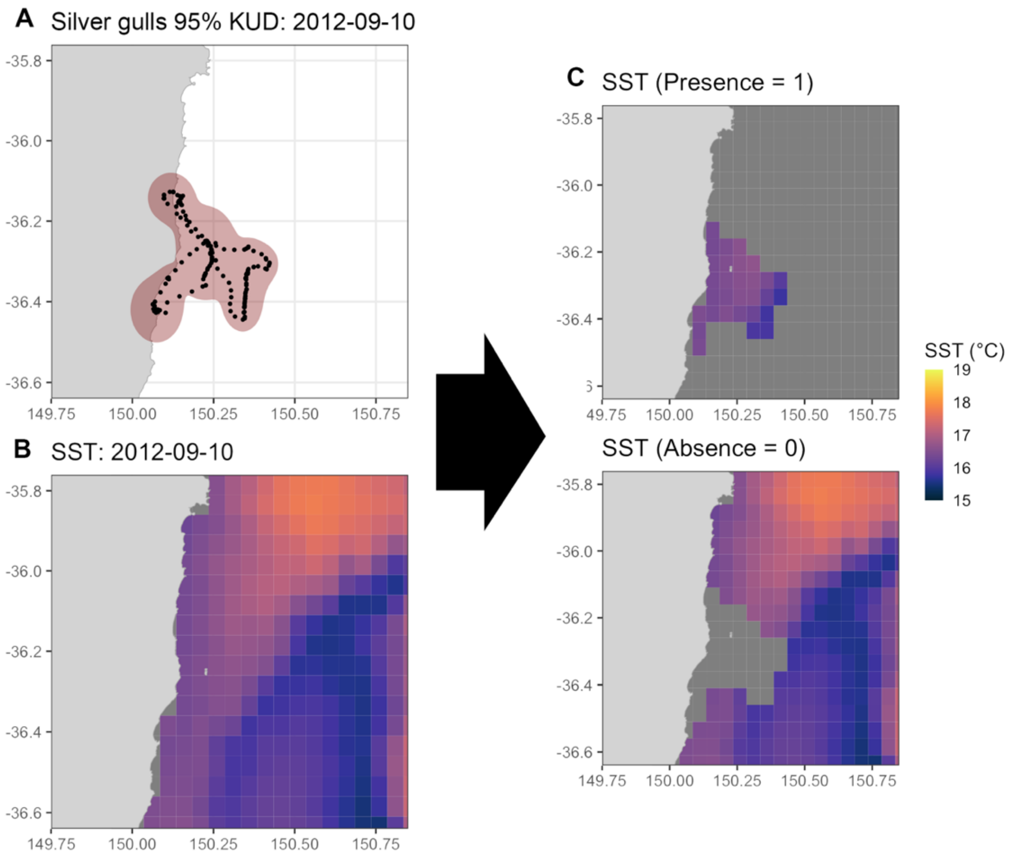

Figure S2. Schematic diagram exemplifying the use of the (A) daily 95% Kernel Utilization Distributions to mask (B) the respective layers of environmental data into (C) presence (area used by the animals) and absence (areas where the animals did not move into) raster layers.

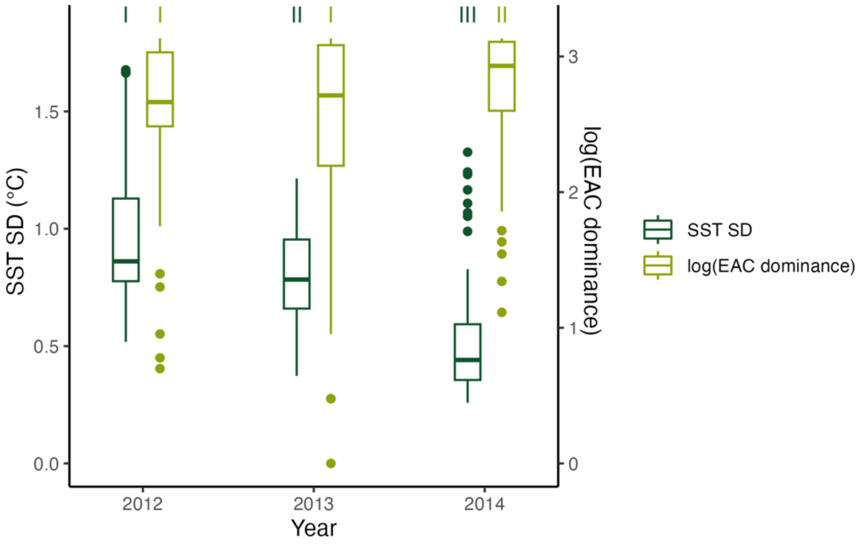

Figure S3. Yearly distributions of oceanographic conditions sea surface temperature standard deviation (SST SD) and the logarithm of number of > 0.5 EAC probability cells [log(EAC > 0.5)] within the study area during the periods of seabird sampling (from 9 September to 5 December). Numbers above boxes (I-III) represent the significant groupings identified with the ANOVA and *post-hoc* Tukey tests.

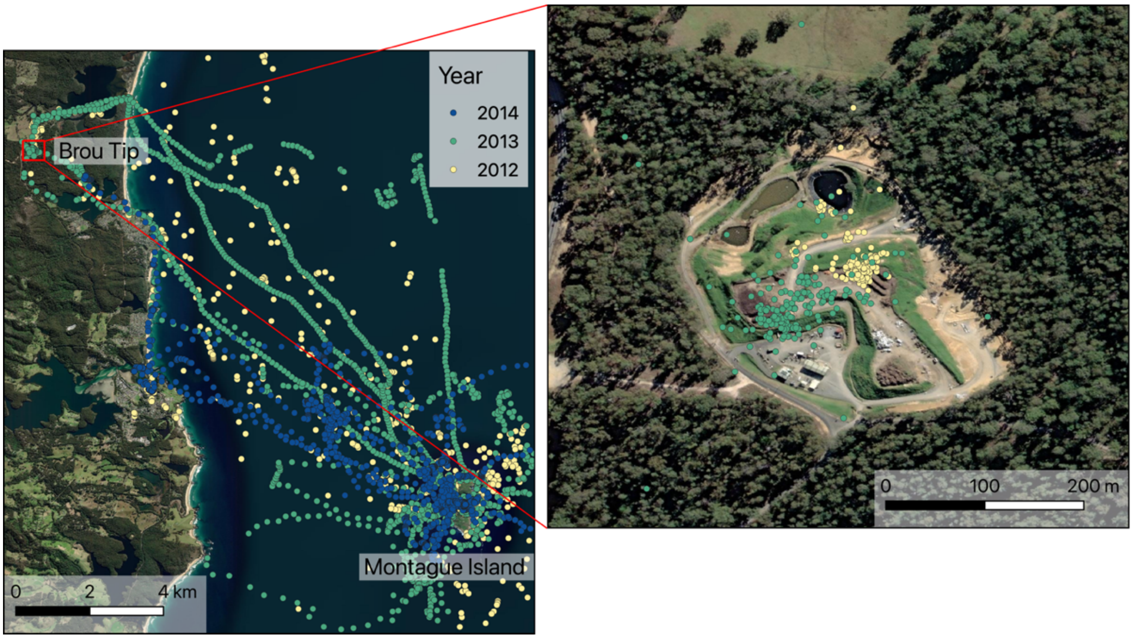

Figure S4. Silver gull tracks by year, showing the location of the local waste management facility (Brou Tip; red inset) used by the species.

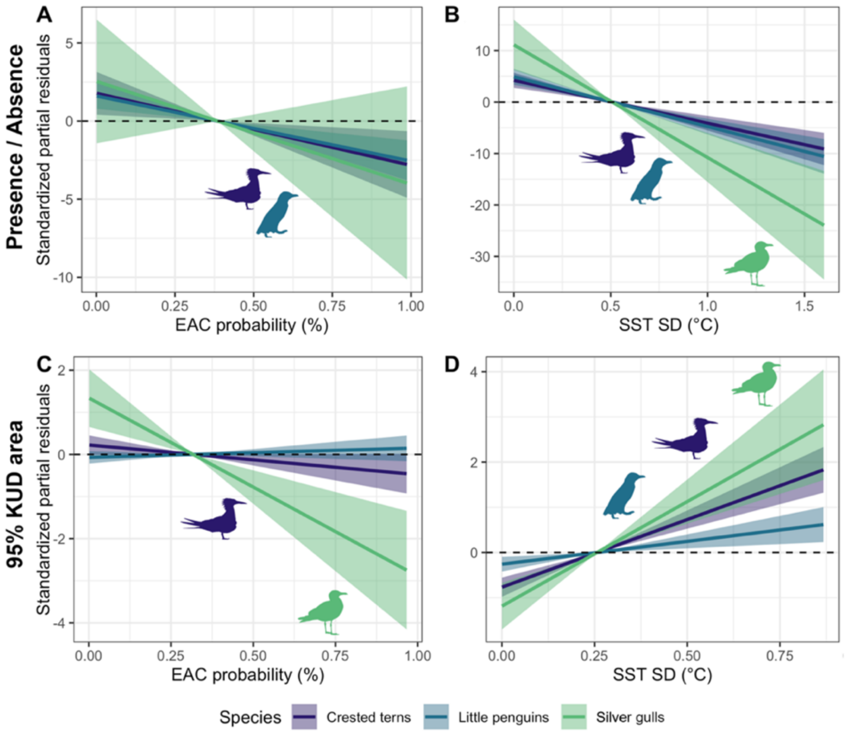

Figure S5. Generalised Linear Mixed Models of daily seabird presence/absence (top panel) and size of daily 95% Kernel Utilization Distribution (KUD) areas (bottom panel), including the significant effects of (A, C) East Australian Current (EAC) probability, and (B, D) sea surface temperature standard deviation (SST SD). Horizontal dashed lines and shaded areas represent the null effects and 95% confidence intervals, respectively. Seabird outlines represent the species with corresponding significant outputs from each model and variable (Table 2).

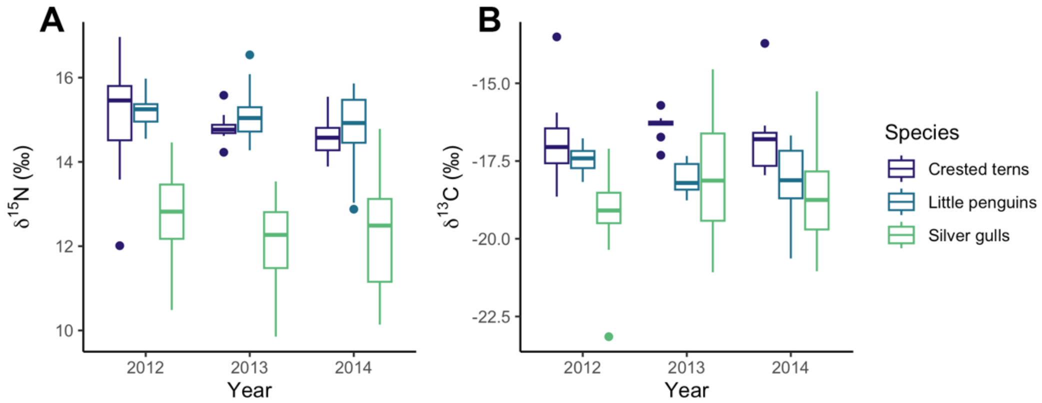

Figure S6. Yearly variations in (A) δ15N and (B) δ13C values per seabird species.
